# International summit on the nutrition of adolescent girls and young women: consensus statement

**DOI:** 10.1111/nyas.13417

**Published:** 2017-07-19

**Authors:** Nancy Krebs, Susan Bagby, Zulfiqar A. Bhutta, Kathryn Dewey, Caroline Fall, Fred Gregory, William Hay, Lisa Rhuman, Christine Wallace Caldwell, Kent L. Thornburg

**Affiliations:** ^1^ Department of Food Science & Human Nutrition University of Colorado School of Medicine Aurora Colorado; ^2^ Division of Nephrology and Hypertension, Department of Medicine Oregon Health & Science University Portland Oregon; ^3^ Centre for Global Child Health The Hospital for Sick Children Toronto Ontario Canada; ^4^ Department of Nutritional Sciences University of Toronto Toronto Ontario Canada; ^5^ Center of Excellence in Women and Child Health the Aga Khan University Karachi Pakistan; ^6^ Department of Nutrition, Program in International and Community Nutrition University of California Davis Davis California; ^7^ Department of International Pediatric Epidemiology University of Southampton Southampton United Kingdom; ^8^ Bob and Charlee Moore Institute for Nutrition & Wellness Oregon Health & Science University Portland Oregon; ^9^ Department of Pediatrics Perinatal Research Center University of Colorado School of Medicine Aurora Colorado; ^10^ Department of Medicine Knight Cardiovascular Institute, Center for Developmental Health, Moore Institute for Nutrition & Wellness Oregon Health & Science University Portland Oregon; ^11^ Catalysis LLC Portland Oregon

**Keywords:** adolescent girls, nutrition, early marriage, food availability, education for girls, transgenerational

## Abstract

An international summit focusing on the difficult challenge of providing adequate nutrition for adolescent girls and young women in low‐ and middle‐income countries was held in Portland, Oregon in 2015. Sixty‐seven delegates from 17 countries agreed on a series of recommendations that would make progress toward improving the nutritional status of girls and young women in countries where their access to nutrition is compromised. Delegate recommendations include: (1) elevate the urgency of nutrition for girls and young women to a high international priority, (2) raise the social status of girls and young women in all regions of the world, (3) identify major knowledge gaps in the biology of adolescence that could be filled by robust research efforts, (4) and improve access to nutrient‐rich foods for girls and young women. Attention to these recommendations would improve the health of young women in all nations of the world.

## Introduction

Sixty‐seven delegates from 17 countries gathered in Portland, Oregon, USA for the International Summit on the Nutrition of Adolescent Girls and Young Women. The meeting brought together scientists with expertise in nutrition and childhood growth, along with field program directors to consider the obstacles preventing adequate nutrition of girls and young women in developing countries. Delegates represented leading scientific institutions, international aid agencies, and government organizations.

The goal of the Summit was to create a dialogue between scientists, program implementers, and policy experts to find innovative remedies for the poor nutrition among adolescent girls and young women in low‐ and middle‐income countries. Historically, interactions between scientists and program implementers have been infrequent, resulting in limited opportunities for translation of advances in nutrition research that could benefit programs serving undernourished populations of girls and young women. The experiences shared by program implementers at the Summit were very important in ensuring that recommendations were meaningful, cost‐effective, and practical.

## Effects of maternal and child undernutrition

Maternal and child undernutrition is the underlying cause of some 3.5 million deaths annually and accounts for more than a third of the disease burden in children younger than 5. In addition, undernutrition has intergenerational effects and as a result, countries and populations with higher rates of maternal and child undernutrition also face deleterious impacts on population and workforce health.^1^, ^2^ We now understand that rates of chronic diseases like diabetes and heart disease can be reduced in future generations if women have access to a nutritious diet before, during, and after pregnancy. Research shows that the origins of many chronic diseases begin during development in the womb and continue during the first few years following conception.^3^, ^4^

Poor nutrition affects more than half of women in low‐ to middle‐income countries, with anemia averaging 40%.^5^ If girls enter the reproductive cycle in a malnourished state, the cycle of maternal malnutrition, fetal growth restriction, infant/child growth faltering, and blunted lifetime productivity is perpetuated. Thus, it is not possible to have a healthy population without adequate nutrition for girls and young women, before, during, and after pregnancy. Years of research have demonstrated the efficacious role of good nutrition in improving the health of mothers and their offspring.^6^, ^7^ Emerging evidence suggests that the health benefits of improved nutrition are particularly robust for adolescent girls.^8^, ^9^, ^10^ In contrast, pregnancy in early adolescence adversely affects fetal development, leading to low birth weight and preterm birth, and negatively impacts a woman's development if she becomes pregnant before she is fully grown.^11^, ^12^

Poor nutrition for girls and young women also has implications for breastfeeding. Exclusive breastfeeding during the first 6 months of life is recommended by the World Health Organization because it reduces the infant's risk of diarrhea, pneumonia, and mortality from other causes.^13^ To ensure optimal nutrient concentrations in breast milk, the mother needs to be well nourished throughout the course of pregnancy and lactation.^14^ This highlights the need for sufficient diets for all pregnant and lactating women, especially adolescents and young women with their own competing growth and development needs.

## Gaps in policies and interventions

According to a recent *Save the Children* report, the majority of nutrition policies and interventions in developing countries target pregnant and nursing women and children up to age 2. Adolescent girls are often overlooked and their nutritional status has not been well studied. In order to break the intergenerational cycle of malnutrition in many populations, more attention needs to be directed toward adolescent girls. Thus, evidence‐based recommendations for programs and interventions for girls are urgently needed.^15^, ^16^

An important knowledge gap includes the efficacy of interventions such as healthy, nutrient‐rich diets, food supplements, and multivitamin‐mineral supplements during the preconceptional period. Studies are also needed to determine the relationship between prepregnancy body size and composition and maternal, neonatal, and child health outcomes^6^ Research scientists and program experts must work together to develop strategic biomedical and implementation research programs that strengthen the evidence base to improve the nutrition of adolescent girls and pregnant and breastfeeding women.

Recognizing the critical gaps between scientific knowledge and field practice, delegates discussed current research findings and identified the primary barriers and critical action steps needed to improve the health of adolescent girls and young women. Participants developed consensus around research and programmatic recommendations to address:

- Major gaps in knowledge on the biology of nutrition and pregnancy as related to adolescent girls, women, and their offspring.
- Major systemic, policy, cultural, and environmental barriers in the achievement of improved nutritional health for adolescent girls, women, and their offspring.

A consensus emerged among the delegates around four overarching themes to address these gaps, barriers, and opportunities. Each broad recommendation is supported by a set of recommended action steps.

## Overarching recommendations

1. *Elevate the urgency of poor nutrition among adolescent girls and young women to a high international priority*
  - Introduce adolescent nutrition as a high priority on global health platforms and agendas, including those that pertain to education, family planning, reproductive health, maternal and child nutrition and health, and food production systems.
  - Demonstrate, from the local up through the national, regional, and global levels, that investing in nutrition for girls and young women is effective and has profoundly beneficial societal and economic implications.
  - Increase investment by donors and governments in programs to address the nutritional needs of adolescent girls and young women.
  - Create opportunities at the local level to improve understanding of the intergenerational and societal implications of healthy nutrition for adolescent girls and young women.
    ○ Develop programs specifically designed to engage in dialogue with diverse community stakeholders and gatekeepers to address gender inequities in nutrition, including girls and young women in the discussions.
    ○ Review and optimize various mechanisms to promote nutrition and health among adolescent girls, including social media, schools, safe spaces, and family support systems.
    ○ Proactively identify and extend efforts to educate and support at‐risk populations on the importance of food quality and nutrition.
2. *Raise the visibility, social status, and health status of adolescent girls around the world*
  Adolescent girls are virtually invisible in many areas of the world. Raising girls’ level of education, health, and social status has potential for generating positive impacts on the economic well‐being of countries. More robust data on the nutritional health of adolescent girls are needed, including documentation of current status, systematic evaluation of programs designed to improve the status of adolescent girls, and ultimately, reduction of the multigenerational harmful effects of poor nutrition and pregnancy in adolescence.
  A number of actions need to be taken to raise the status and improve the health outcomes of adolescent girls, young women, and their offspring:
  - Define the indicators of good adolescent health and nutrition. This will require better population‐based data collection by program implementers and local governments.
  - Identify noninvasive, inexpensive biomarkers for nutrition and comprehensive components of health to determine the prevalence of nutritional deficiencies in adolescent girls and young women and to evaluate interventions for this group.
  - Develop sensitive composite indicators that reflect overall well‐being (physical, mental, and emotional).
  - Collect anthropometric data (height, weight, and body mass index (BMI), which is a person's weight (in kilograms) over their height squared (in centimeters)) for adolescents in surveillance systems and as outcomes for interventions.
  - Initiate high priority programs to educate all girls of school age and to keep them in school through their teen years.
  - Reduce societal acceptance of and complicity in child and early adolescent marriage.
  - Establish new programs designed to prevent pregnancies in early adolescence (ages 10–15) to avoid the many deleterious outcomes for girls, their offspring, and future generations.
  - Delay pregnancy beyond late adolescence (ages 16–19), and increase interpregnancy intervals.
  - Enlist the participation and voices of adolescent girls and young women in each step of health and development improvement programs. This will allow program directors to better understand the drivers of their behavior and to create intervention strategies that will resonate with them.
3. *Address knowledge gaps in the biology of adolescence and define appropriate nutrition*
  The biologic impact of compromised nutrition among girls and young women is poorly understood though it is clear that there are both immediate and long‐term detrimental effects. Thus, the potential health and societal benefits of improved nutrition for girls are enormous. Basic research scientists and program experts must work together to develop a strategic biomedical research agenda on adolescent health, pregnancy, and lactation. Such an effort would identify the nutritional drivers that underlie effective preventive interventions.
  - Devise and prioritize a research agenda to address knowledge gaps in the nutrition‐sensitive development of adolescent girls and young women, including:
    ○ Extent of plasticity and potential for recovery from past nutritional and health insults, including examination of microbiomic and epigenetic changes in response to diet and nutrition improvement interventions.
    ○ Identification of critical windows for the most effective intervention outcomes.
    ○ Interactions and interdependence among diet, nutrition, infection/inflammation, social stress, growth, and development.
    ○ Impact of improved nutritional status during adolescence on long‐term health and neurodevelopmental outcomes.
    ○ Physiological impacts of adolescent pregnancy and dietary mitigation of detrimental effects on mother and baby.
  - Evaluate current specific nutrition interventions in adolescent girls and young women, including balanced energy protein supplements, micronutrients, small quantity lipid‐based nutrient supplements, and food‐based strategies for effectiveness at all stages of the reproductive health continuum: preconceptional, prenatal, and postnatal maternal health and long‐term outcomes.
  - Rigorously evaluate programmatic approaches for nutrition interventions to target and reach adolescent girls and young women and share best practices rapidly across delivery platforms such as schools, health centers, youth and women's groups, and civic and community organizations.
  - Develop protocols for sharing evidence‐based practices that can be tailored to diverse contexts, accounting for factors that can vary by region, for example, food availability and storage; climate and seasonal considerations; disease prevalence and patterns; access to clean water; and community, religious, and cultural practices.
4. *Improve nutritional health of adolescent girls and young women, and their offspring*
  - Encourage local programs that improve access to a variety of affordable, nutritious foods in adequate quantities to meet the relatively high nutrient needs of the adolescent. Replicate programs that have demonstrated effectiveness.
  - Broadly institute evidence‐based, effective nutritional, supplemental, and health interventions across the adolescent developmental continuum.
  - Improve food quality to minimize exposure to disease and disability causing toxic factors, including herbicides and pesticides, plant and fungal toxins, and antinutrients.
  - Educate community and government leaders on the special nutritional needs of pregnant and lactating adolescent girls and young women and the health benefits derived from the provision of healthy foods over the course of pregnancy and lactation.
  - Engage multiple stakeholders in communities, including households and private/civil society sectors, to recognize and support achievement of the far‐reaching benefits of enhanced nutritional health of adolescents.
  - Work with a broad set of partners (including economic development organizations, agriculture industry, local food producers, and civic and government leaders) to make nutrient‐rich foods more affordable, accessible, and locally grown.

## Conclusion

Addressing the concerns articulated in this consensus statement will require that research scientists, health care providers, policy‐makers, nutrition program implementers, and community leaders collaborate to ensure that the latest research can be translated into effective local interventions. While the task is daunting, the return on investment to elevate the status of adolescent girls around the world would yield profound health and economic improvements for generations to come. A number of global and regional movements are working to increase the empowerment of girls. Those movements have at their heart the recognition that societal health and economic development are linked to the health and well‐being of girls and young women.

If government and community programs would act upon these recommendations, the health of populations around the world would be dramatically improved across multiple generations. As Dr. Margaret Chan, Director‐General of the World Health Organization, described, “We can reduce maternal anaemia, low birth weight and child stunting and bring down the risk of noncommunicable diseases within a generation. We can achieve this by giving nutrition the attention it deserves.”^17^ This goal cannot be achieved unless the health and nutritional well‐being of adolescents and young women is improved overall, prior to conception, and during and after pregnancy. This document recommends bold actions because the need is urgent and the impact of positive action would improve the health of future generations.

## Competing interests

The authors declare no competing interests.
